# The informed consent process in health research with under-served populations: a realist review protocol

**DOI:** 10.1186/s13643-021-01652-2

**Published:** 2021-04-09

**Authors:** Eleanor Hoverd, Sophie Staniszewska, Jeremy Dale

**Affiliations:** 1 CRN West Midlands Primary Care/Unit of Academic Primary Care, Warwick Medical School, Warwick University, Gibbet Hill Rd, Coventry, CV4 7AL England; 2 Warwick Research in Nursing, Warwick Medical School, Warwick University, Coventry, England; 3 Unit of Academic Primary Care, Warwick Medical School, Warwick University, Coventry, England

**Keywords:** Informed consent, Under-served, Health research, Realist review

## Abstract

**Background:**

The informed consent process aims to provide potential participants with information about health research that enables them to make an informed decision as to whether they choose to participate, or not. However, it remains unclear as to whether the process is effective for those who are under-served in health research. It is a pivotal issue within health research that the diversity of people who participate is broadened. The National Institute for Health Research (NIHR) pledges to support equality, diversity and inclusion, actively creating opportunities for all citizens whom are eligible, to take part in health research.

**Methods:**

In order to understand how the informed consent process for under-served populations in health research works, under what circumstances and in what respects, a realist review approach will be undertaken. Searches will be carried out using electronic databases (EMBASE, MEDLINE, Web of Science and PsycINFO), along with selected websites and grey literature. Development of initial rough programme theory(ies) will lead to a more refined programme theory that will provide an explanation of context, mechanism and outcomes. Stakeholder involvement by NIHR (Public) Research Champions, health professionals and clinical academics will provide expert opinion about concepts and programme theory.

**Discussion:**

Findings of this realist review will highlight how the informed consent process in health research affects the experience and decision-making process of potential participants from under-served populations. They will be written up in accordance with RAMESES guidelines and disseminated to patients and the public, health researchers, health professionals and policymakers through peer-reviewed publication, presentations and discussions. The review will contribute to our understanding of the mechanisms that cause both positive and negative outcomes in the informed consent process for those whom are often under-represented in health research to inform policy, study design and delivery.

**Supplementary Information:**

The online version contains supplementary material available at 10.1186/s13643-021-01652-2.

## Background

Health research in the twenty-first century must be inclusive in order to generate evidence that is applicable to diverse populations [1]. However, it is recognised that many groups are under-served in health research. There is an increasingly urgent need to address this. One factor contributing to this is the informed consent process. Through a lack of flexibility and sensitivity in the way in which it is designed and delivered [2–4], participants from under-served populations may be inadvertently excluded [5–7].

Defining under-served as a concept is vital to developing understanding of how informed consent enables or inhibits diversity in research. The INCLUDE (Innovations in Clinical Trials Designs in Under-represented groups) project has been looking to improve representation of under-served groups in clinical trials in the UK [8]. Following involvement from a range of stakeholder groups as part of the project, it is recommended that the term under-served is preferred when describing the many groups of people whom are often not represented in health research, such as ethnic minority groups [8]. It is unclear from the wider literature as to what a suitable definition of under-served groups is, as this varies according to setting and perspective, but they are often those groups whom are met with difficulties in accessing and utilising resources [9]. It is a term frequently used by the World Health Organization (WHO) often describing areas as rural, remote or poor and populations as excluded or vulnerable, although a definition is not evident [10, 11]. This will be explored further during the realist review as part of mapping out the territory, identifying the types of populations that are under-served in health research, as part of the scoping the literature [12]. In addition, a suitable working definition will be proposed by the stakeholder groups for the purpose of this review.

Previous studies on the informed consent process have reported that potential participants’ lack of understanding of study information is a significant issue [13–15]. The informed consent process, when carried out effectively, respects the decision made by a potential participant as to whether they wish to take part, or not, in health research [16]. However, evidence suggests that information provided to potential participants is often complicated and lengthy causing concern around issues of comprehension and effectiveness, putting into question the validity of the whole process [3, 16–19]. This raises questions about the effectiveness and appropriateness of the informed consent process—the way in which it is designed and delivered for populations that may not be suited to receiving a uniform process and about the circumstances under which it works, if it works [4, 5].

Previous systematic reviews have focussed on understanding and improving the informed consent process, although little has changed in the way in which it is carried out [13, 18, 20]. There is some evidence to suggest that the use of multi-media interventions to improve understanding, along with extended discussions during the informed consent process, may be effective, although these interventions have not been adopted in practice [18, 21]. In the past, there have been a number of concerns from researchers and Institutional Review Boards (IRBs) including how to conduct appraisal of computer-based informed consent, costs associated with the development of new technology to support the informed consent process, lack of support from government agencies and issues around protecting confidentiality, thus resulting in reluctance to change from traditional consent methods [15, 22].

Since these systematic reviews were carried out, global healthcare has rapidly begun to adopt digital technology to deliver interventions, provide information and communicate to patients [23]. The World Health Organization (WHO) has recently produced a Global Digital Health Strategy 2020–2024, aiming to make health systems around the world stronger, for the benefit of all populations [24]. The need for further research to understand how the informed consent process is experienced and understood by those whom are under-served is vital in order to contribute to the development of a more flexible, innovative informed consent process. This may also provide further benefit in understanding the requirements of these populations in order to develop a digital process that has the capability of being utilised more broadly within health care [18].

Failing to include diverse representation of populations in health research risks producing findings that are not applicable, nor meaningful to the wider population, can lead to methodological weakness [25]. It can also limit discovery and produce unforeseen consequences, preventing the broader population from benefitting [26]. The recent effect of COVID-19 on ethnic minority populations demonstrates the urgent need for ensuring health research is inclusive [22], providing an opportunity to identify how research processes are supporting this requirement. There is a need for further evidence to help policymakers and health researchers improve the process to make health research more inclusive, keep up with the diverse needs of our population, improve our understanding of what works and for which groups, and adapt it to work in various settings and under different conditions, whilst maintaining its validity.

### Realist review methodology

The informed consent process is complex [13, 17, 18, 27]. A realist review will advance knowledge by offering a deeper understanding as to how, why and in what contexts an informed consent process works best for enabling under-served populations to make an informed decision as to whether to participate in health research [28]. Whilst there is considerable literature on the informed consent process in health research, there remains a gap in addressing the complexities of the process that affect the opportunity for under-served individuals to make a decision about taking part.

This realist review will gather feedback and advice from content experts on the evolving programme theory and will not entail primary data collection. NIHR (Public) Research Champions and health professionals will be asked to offer their views as ‘content’ experts concerning the believability and comprehensiveness of a programme theory and offer suggestions of locating further data [29]. Stakeholders will provide expert opinion, adding value to the review [28]. This iterative process will add to the evidence in a way that goes beyond a traditional systematic review [30]. The experiences of these groups will help to clarify how the informed consent process is expected to work, whilst including and referring to the evidence in order to explain the mechanisms.

Realist reviews seek to develop theories using an explanatory approach which is particularly important for understanding informed consent as a complex social intervention that heavily depends on the context of delivery and how it is delivered [31]. The CMO configurations (C—context, M—mechanism, O—outcome) embedded within the programme theory will help to provide causal explanations that explain the relationships between contexts (C), which ‘activate’ mechanisms, resulting in one or more outcomes (O) [32]. This will lead to the development of a programme theory that will be tested and refined, providing an explanation as to how the informed consent process operates [12].

An important aspect of the realist review is its ability to provide a transferable framework that facilitates consideration of how a process such as informed consent is controlled and understood by government authorities; in the UK, these include the Medicines and Healthcare products Regulatory Agency (MHRA) and Health Research Authority (HRA), IRBs, researchers, health professionals and participants in health research [31]. The explanatory powers of the programme theory of informed consent are transferable to different settings where the same mechanisms are likely to be in operation. This review will also provide evidence around what the current informed process experience looks like for under-served populations and, in addition, will explore which mechanisms, contexts and conditions affect their decision-making during the informed consent process.

This realist review protocol has been written according to the PRISMA-P guidelines [33].

## Methods

### Review aim, questions and objectives

#### Over-arching research question:

What contextual factors influence the decision to take part in health research in under-served populations?

#### Aim

The aim is to develop and refine a programme theory that explains the factors that influence the decision to take part in health research in under-served populations.

#### Objectives

1. To undertake a realist review of the literature to identify the contexts and mechanisms that affect the informed consent process and the decision to participate in health research in under-served populations
2. To draw on the review’s programme theory to develop guidance that addresses barriers to participating in health research in under-served populations

### Stakeholder groups

This review will involve stakeholders (described below) who will be invited to comment on the process of informed consent in health research as NIHR (Public) Research Champions, health professionals and clinical academics. NIHR (Public) Research Champions are volunteers who are interested in raising awareness about health research, particularly to those who often do not participate [34]. This mix of stakeholders is considered to hold positions that are relevant to the topic of the review and to have a vested interest on behalf of under-served populations [35]. Ethical approval is not required as stakeholders will be contributing collaboratively to content and dissemination, not providing data [36–38]. These stakeholders will provide advice on what may be in a programme theory, helping with refinement and sharing views on the credibility of a programme theory, along with suggestions as to where to find more data [29]. They will be considered “content” experts as entitled by Wong and will be invited to be involved and asked to provide input at certain steps in the review as displayed in Table 1 [29]. It will be beneficial to share the findings with health researchers and policymakers such as the National Institute for Health Research (NIHR) and Health Research Authority (HRA), but it is not possible for the scope of this review to involve them in discussions as this would require more time and resources to do so.

**Table 1:** Stakeholder input

Invitation via email will be to the following potential stakeholders to join a realist review discussion group via videoconferencing:	
- Staff from the Clinical Research Network (CRN) West Midlands that have experience of working within a health research setting - Clinical academics from the Clinical Trials Unit, Warwick Medical School - Health professionals in the West Midlands from a secondary care setting that have experience of delivering health research - NIHR (Public) Research Champions whom are part of the Clinical Research Network West Midlands Equality, Diversity and Inclusion working group	
There will be two separate groups, one each for professionals and NIHR (Public) Research Champions, who will meet virtually, up to three times during the following points:	
- At the second iteration: meeting to develop a working definition for the term under-served and to prioritise programme theory(ies) - At the third iteration: meeting to refine programme theory - End of project final meeting when review is complete	

Due to the current social distancing requirements associated with the COVID-19 pandemic, discussions with stakeholders will be held using the Microsoft Teams online platform unless stakeholders indicate a preference for other technology that would support accessibility [39]. Stakeholders will be sent an invitation to join the meeting via EH, to limit accessibility to those invited. There will be two, separate stakeholder group meetings, one for health professionals/clinical academics and one for NIHR (Public) Research Champions, that will enable uninhibited discussions [35]. A maximum of 10 participants per meeting will be set to provide the opportunity for all to speak. Stakeholders will be contacted via email before the scheduled meetings to offer a brief training session on Microsoft Teams (if required) and a summary of what to expect to ensure meetings run smoothly.

### Patient and public involvement

The initial ideas for this review were discussed with NIHR (Public) Research Champions at a local, West Midlands primary care Patient Public Involvement (PPI) meeting held by EH. It was agreed that the topic is important and relevant to patients and the public. NIHR (Public) Research Champions will form a key stakeholder group and will be central to providing expertise at the second and third iterations of the review. At the end of the review, they will comment on the findings and contribute to the dissemination plan through helping to write a lay summary and designing a leaflet for dissemination to their peers.

Patient and public involvement through NIHR (Public) Research Champions will provide insights and opinion on the informed consent process including what it should look like in health research, along with sharing valuable input throughout the realist review steps [38]. PPI will be facilitated through inviting an existing Equality, Diversity and Inclusion working group from the Clinical Research Network West Midlands that consists of NIHR (Public) Research Champions who are interested in tackling health inequalities within health research specifically. In addition to the NIHR (Public) Research Champions, snowballing of interest from other PPI volunteers who express interest in providing expertise will be invited at their request, to comment at planned meetings also.

### Multi-disciplinary involvement

In addition to the PPI stakeholder group, a multi-disciplinary group of health care professionals, clinical academics and the PPI Lead of the CRN West Midlands Equality, Diversity and Inclusion working group from the West Midlands will be invited for consultation, drawing on their expertise and insider knowledge related to the informed consent process in health research and under-served populations. The group collectively will have a variety of experiences including carrying out the informed consent process with local populations from all across the West Midlands, including rural and urban areas and in various settings, such as care homes, secondary care settings and general practice; experience of recruiting participants from under-served populations; and a vested “stake” in reducing health inequalities in health research and also insight into the difficulties of recruiting potential participants from under-served populations in health research. This experience and knowledge will be beneficial when prioritising, refining programme theory and building CMO (Context, Mechanism, Outcome) configurations [29]. Some professionals may have experience of not being able to carry out the informed consent process with particular patients, or in certain locations, and these experiences are also considered vital in helping to provide explanations of what works and what does not, for whom and in what context.

As recommended by Pawson et al. (2005), a series of steps in accordance with standard Cochrane headings can provide a helpful approach to carrying out a realist review. Therefore, this review will be conducted in this logical way [31].

#### Step 1 Development of an initial programme theory

In order to develop an initial programme theory that will ‘map out’ what the informed consent process involves and how it is predicted to work, a scoping search will be conducted [40]. It is recognised that it is natural for a scoping search to be an iterative process and often challenging, due to the complexity of the intervention under study [41]. This may require refining of the research question(s) for a length of time during the review [42]. EH will carry out the scoping search based on terms focussed on the intervention (informed consent). This will enable identification of existing theories about how under-served populations experience and understand information provided during the informed consent process in health research and in what context, and under what conditions it is effective, or ineffective. A combination of electronic databases, along with websites such as the Health Research Authority (HRA), will be searched, see Table 2. It is also acknowledged that through discussions with stakeholders, further sources may be identified and used to inform the programme theory. The support of an information specialist to support searching will also be requested, as recommended by Pawson [32].

**Table 2:** Data sources

Type of source	Name of source(s)
Electronic databases	EMBASE, MEDLINE, Web of Science, PsycINFO
Websites	UCL IRIS (Institutional Research Information Service), HRA, MHRA, NIHR, (formerly INVOLVE), legislation.gov.uk
Grey literature	Search engines (Google), Editorials, Opinion pieces
Stakeholders	E.g. reports, conference presentations

A list of important intervention theories for exploration will be compiled that will be of relevance to the research questions. Following the creation of a list of all relevant programme theories, they will be shortlisted according to which are deemed to be the most important for further exploration [31].

#### Step 2 Evidence searching

Purposive sampling will be used to search the literature. Using this sampling method will enable an iterative process that will allow repeated searching and refinement of search terms as understanding develops throughout the review [31]. Pawson et al. suggest approaching the search in four parts as follows:

1. Carry out a background search of the literature
2. Develop, refine, verify or disprove a theory based on available evidence
3. Implement the search strategy to identify relevant evidence
4. Perform a final search that will help to identify further studies that may contribute to sharpening the programme theories discovered [31].

Free text and MeSH (medical subject headings) will be used to ensure thoroughness. Each database will require an individual search which will be fine-tuned accordingly, to ensure relevant papers are identified. The reference lists of papers included will be examined for locating further papers that may add to the richness of data. To ensure validity and diligence of the search process, experts within the field of interest will be contacted to verify results. Following each stage of the search, it will be essential to check whether any new literature adds anything different to the knowledge around the intervention (informed consent process). This will help to determine when saturation has been reached [31].

Searching will be conducted and reported in accordance with RAMESES standards [38]. An example of an initial search strategy for MEDLINE, containing the search terms, is available in Additional file 1 (Search 1).

#### Step 3 Document selection and data extraction

Preliminary screening of the literature using article titles and abstracts will identify all papers that are potentially relevant. Secondary screening of full texts by the main reviewer (EH) will be carried out according to pre-determined inclusion/exclusion criteria (see Table 3). A second reviewer will screen a 10% sample of the results, for agreement, with a record of exclusions made throughout the screening process. Any disagreements will be settled by the stakeholder group. Records will be managed using *Endnote Online* to manage references.

**Table 3:** Inclusion/exclusion criteria

Inclusion criteria	
- Quantitative and qualitative studies, grey literature, websites, stakeholder recommendations (e.g. reports, conference papers). - The search will be limited to a 15-year publication period (2005–2020) to provide a large enough overview of relevant literature. - All studies that report on experiences and decision-making involved in the - informed consent process in under-served populations will be included. - Any source of data that is deemed relevant by the stakeholder group. - Literature that explores the concept and practice of informed consent.	
Exclusion criteria	
- Studies or sources of data that are not relevant to the informed consent process in health research in relation to under-represented populations.	

According to Wong, realist reviews must take a logical approach in considering the trustworthiness of data [29]. It is important to note that because this review is developing theory and data will come from many sources, Wong suggests assessing the trustworthiness of data by [29]:

- Presuming that empirical data has been acquired methodically.
- Deciding if there is lack of clarity around data collection methods, in which case trustworthiness should be regarded with uncertainty.
- Making the effort to locate multiple, pertinent sources of data in relation to facets of programme theory. Relevance is key [29].

In addition to this, utilising a data extraction form will help to provide a reliable approach to data extraction [42]. The data extraction form will be developed once a programme theory has been established as the content of the form will be informed by the initial programme theory. The data extraction form will be piloted on two articles before implementation, but an open-minded approach will be taken as it is understood that it may be necessary to have several forms to avoid a one-size-fits all approach to extraction [28].

Data extraction forms will be used in conjunction with Pawson et al.’s recommended approach to gathering information through notetaking and annotation [31, 43–46]. Utilising these methods will provide a comprehensive and auditable trail for data extraction and critical appraisal.

#### Step 4 Data analysis and synthesis

Synthesis of the evidence will aim to develop, test and refine the programme theory through analysis of the data [47]. Data will be mapped to create a data matrix according to CMO configurations which will help with interpretations and in producing theory [38]. Data synthesis will be guided by the approach used by Rivas et al. to support interpretation and development of the programme theory. Following a similar approach will improve methodological quality through [43]:

- Comparing evidence to gain better understanding
- Amalgamating data that provide evidence of corresponding mechanisms and outcomes
- Further reflecting on evidence that demonstrates opposing outcomes, but similar contexts
- Considering the circumstances of evidence that finds different contexts and outcomes
- Using judgement to determine methodological strengths and weaknesses [30, 48]

#### Step 5 Refinement and validation of the programme theory

Refining and validating the programme theory that is based on the synthesised data will be carried out with stakeholders who will provide ‘expertise’ to ensure their knowledge and experiences support it [28]. EH will conduct a remote meeting via Microsoft Teams to share analysis and results of the review allowing stakeholders to be involved in interpreting and refining the programme theory. The involvement of stakeholders will improve how the findings will assist with creating recommendations. Any matters raised by stakeholders that have not been discovered in the findings will be further explored to confirm, refute or refine the programme theory. The timescale for completion of the review will limit the number of times the programme theory can be refined. The final programme theory will be presented in a narrative form with a description of the realist synthesis, in accordance with RAMESES guidelines and standards [38].

#### Step 6 Development of recommendations

The review is intended to provide government agencies, IRBs, researchers and health professionals with an explanation of what is effective, what is ineffective and what is unknown about the informed consent process for improving experience and understanding to aid decision-making in under-served populations. EH will lead a discussion remotely whereby stakeholders will be presented with the final programme theory and be given time to determine and agree upon a set of recommendations. Input from the identified key stakeholders will ensure relevance of the recommendations. They may also contribute to further recommendations for developing training on how best to deliver the informed consent process and highlight potential avenues for exploring the design of health research studies.

### Ethics and dissemination

Ethical approval is not necessary for this study although all stakeholders will be fully informed of the study intentions. However, there are several ethical considerations to make around patient and public involvement as recommended by Pandya-Wood et al. [19]. Some of these ethical considerations will also be applied to the professional stakeholder group as to ensure a professional approach is taken. These include role of the public contributor, avoidance of tokenism, the opportunity to withdraw, fairness of opportunity, taking a sensitive approach, and respecting confidentiality and valuing public contributions [19] (see Table 4).

**Table 4:** Ethical considerations for Patient Public Involvement

**Role of public contributors**	The role of the public contributors will be made clear when they are invited to be part in the stakeholder group, through written information with the opportunity to discuss verbally if they wish. Their contributions will include developing a working definition of the term under-served, involvement in refining programme theory providing their ideas as to what is important when considering and refining programme theory, with sufficient time allocated to allow for discussion and flow of ideas.
**Avoidance of tokenism**	Details of PPI contributions and how they will be utilised and shaped programme theory will be described in the review.
**Opportunity to withdraw**	It will be clearly communicated to public contributors that they may withdraw their involvement at any stage in the review process without needing to provide a reason.
**Fairness of opportunity**	The researcher will seek to engage pragmatically, with an already formed group that represents equality, diversity and inclusion. This will also be a limitation, because the scope of this review will not allow more time, or funds to engage with a larger group.
**Sensitive approach**	A sensitive approach will be taken with the public contributors with every effort to ensure personal information is not revealed in relation to individuals that are involved, whilst being aware of any potentially, undesirable reactions of members of the group.
**Confidentiality**	It will be clearly stated to public contributors that should any personal issues be discussed during the meetings, this information will remain confidential.
**Valuing public contributions**	A pragmatic approach will be taken with a view to being flexible with technology, utilising what works best. Regular communication to all public contributors will be carried out with their involvement described within the review and how it was included.

The expertise from NIHR (Public) Research Champions, or public contributors, during stakeholder meetings will help to refine the focus of the searching, help to inform the initial programme theory, provide advice throughout the review, discuss and provide feedback on CMO configurations, and help to create recommendations [29].

#### Dissemination

Findings from the review will be disseminated to patients and the public (in the form of a lay summary), researchers, health professionals and policymakers in relation to the informed consent process with under-served populations. The findings will offer deeper understanding about the relationships between the informed consent process and the contexts and mechanisms which influence decision-making by under-served populations. Findings will be shared as set out in Table 5. The aim of disseminating findings with policymakers is to influence their thinking [28].

**Table 5:** Dissemination plan

Patients and the public	Researchers/health professionals	Policymakers
NIHR Centre for Engagement and Dissemination (CED) (lay summary) Public Involvement and Lay Accountability in Research and innovation PILAR) (lay summary)	Publication in a peer-reviewed journal	Summary of findings emailed to HRA Head of Policy
West Midlands Research Champion Forum (presentation)	Poster at academic conference	Summary via NIHR *Signals and Connect* newsletters
NIHR Patient and Public Involvement and Engagement (PPIE) Google Community (link to lay summary)	Poster Presentation at West Midlands R&D Forum	Email MHRA Director of Inspection, Enforcement and Standards with summary
Twitter (lay summary)	Twitter/ Warwick Medical School Unit of Academic Primary Care webpage (summary of findings)	Twitter (@HRA News, @MHRApress)—link to findings

## Discussion

The UK is currently lacking policy that drives inclusion in health research. This realist review will derive from the available literature, along with the expertise of stakeholders, a refined programme theory to explain the context, mechanism and outcomes for the informed consent process in health research with under-served populations.

To our knowledge, this is the first realist review aimed at developing an original programme theory on the informed consent process in health research for under-served populations. As with any research, there are strengths and limitations. These include:

- Using realist methods to explore the contexts and mechanisms of the complexities behind the informed consent process in health research, for under-served populations
- The involvement of National Institute for Health Research (NIHR) public Research Champions, healthcare professionals and clinical academics in refining the programme theory will ensure the study is relevant
- Availability of evidence relating to under-served populations participating in health research may limit theory building
- Owing to the current COVID-19 pandemic**,** stakeholder involvement in the programme theory development may be restricted, and will be limited to remote communication

The results of the review will be useful for policymakers, health researchers and government agencies—sharing explanations about the informed consent process—for creating a health research environment that is more inclusive.

It is worth noting that the current COVID-19 pandemic may potentially cause disruption to this review. This is most likely to affect stakeholder involvement. A flexible approach to stakeholder meetings aims to minimise this as much as possible. Any impact, or variations to the original protocol because of the pandemic, will be discussed in the review results paper.

## Supplementary Information

**Additional file 1:.** Draft MEDLINE search strategy

## Data Availability

Not applicable.

## Abbreviations

CED: Centre for engagement and dissemination
CMO: Context, mechanism, outcome
CRN: Clinical research network
HRA: Health research authority
INCLUDE: Innovations in clinical trials designs in under-represented groups
IRBs: Institutional review boards
MHRA: Medicines and healthcare products regulatory agency
NIHR: National institute for health research
PPI: Patient and public involvement
PPIE: Patient and public involvement and engagement
WHO: World health Organization

## References

[1] Corbett J, D'Angelo C, Gangitano L, et al. Future of Health: findings from a survey of stakeholders on the future of health and healthcare in England. Dep Health. 2017;1–72. https://www.rand.org/pubs/research_reports/RR2147.html. Accessed 11 Feb 2020. 10.7249/rhq-07-03-01 PMC5873518 29607245

[2] Davis T, Holcombe R, Berkel H (1998). Informed consent for clinical trials: a comparative study of standard versus simplified forms. J Natl Cancer Inst.

[3] Dunn L, Jeste D (2001). Enhancing informed consent for research and treatment. Neuropsychopharmacology.

[4] Kadam R (2017). Informed consent process: a step further towards making it meaningful!. Perspect Clin Res..

[5] Hughson J, Woodward-Kron R, Parker A, et al. A review of approaches to improve participation of culturally and linguistically diverse populations in clinical trials. Trials. 2016. 10.1186/s13063-016-1384-3. 10.1186/s13063-016-1384-3 PMC4880985 27229153

[6] McDougall G, Simpson G, Friend M. Strategies for research recruitment and retention of older adults of racial and ethnic minorities. J Geront Nurs. 2015. 10.3928/00989134-20150325-01. 10.3928/00989134-20150325-01 PMC6415923 25849063

[7] Redwood S, Gill P (2013). Under-representation of minority ethnic groups in research- call for action. Br J Gen Pract.

[8] Witham, M.D., Anderson, E., Carroll, C. et al. Developing a roadmap to improve trial delivery for under-served groups: results from a UK multi-stakeholder process. Trials. 2020; doi.org/10.1186/s13063-020-04613-7 10.1186/s13063-020-04613-7 PMC7395975 32738919

[9] Wang V (2017). Encyclopedia of Strategic Leadership and Management Hershey.

[10] World Health Organisation. Increasing access to health workers in underserved areas: a conceptual framework for measuring results: Bulletin of the World Health Organisation. Geneva: WHO Press; 2010. www.who.int/bulletin/volumes/88/5/09-070920/en/. Accessed June 2020. 10.2471/BLT.09.070920 PMC2865656 20461135

[11] World Health Organisation. Resolution on community health workers to be considered at the upcoming World Health Assembly. News and Events. Geneva: WHO Press; 2019 https://www.who.int/hrh/news/2019/community-health-workers-resolution-at-wha/en/. Accessed June 2020.

[12] Papoutsi C, Mattick K, Pearson M, Brennan N, Briscoe S, Wong G (2017). Social and professional influences on antimicrobial prescribing for doctors-in-training: a realist review. J Antimicrobial Chemo.

[13] Flory J, Emmanuel E (2004). Interventions to improve research participants' understanding in informed consent for research: a systematic review. JAMA.

[14] Montalvo W, Larson E. Participant comprehension of research for which they volunteer: a systematic review. J Nurs Schol. 2014. 10.1111/jnu.12097. 10.1111/jnu.12097 25130209

[15] Pandiya A (2010). Readability and comprehensability of informed consent forms for clinical trials. Perspect Clin Res..

[16] HRA. Applying a proportionate approach to the process of seeking consent. 2017. http://www.file:///C:/Users/mhsman/Downloads/Proportionate_approach_to_seeking_consent_HRA_Guidance.pdf. Accessed: 20 Apr 2020.

[17] Joffe S, Cook E, Cleary P (2001). Quality of informed consent in cancer clinical trials: a cross-sectional survey. Lancet.

[18] Nishimura A, Carey J, Erwin P. Improving understanding in the research informed consent process: a systematic review of 54 interventions tested in randomised control trials. BMC Med Ethics. 2013;14(1). 10.1186/1472-6939-14-28. 10.1186/1472-6939-14-28 PMC3733934 23879694

[19] Pandya-Wood R, Barron D, Elliot J (2017). A framework for public involvement at the design stage of NHS health and social care research: time to develop ethically conscious standards. Res Involvement Engagement.

[20] Thanh Tam N, Tien Huy N, Bich Thoa L (2015). Participants' understanding of informed consent in clinical trials over three decades: systematic review and meta-analysis. Bull World Health Organisation.

[21] Jimison H, Sher P, Appleyard R (1998). The use of multimedia in the informed consent process. J Amer Med Info Assoc.

[22] Kirby T (2020). Evidence mounts on the disproportionate effect of COVID-19 on ethnic minorities. Lancet Respir Med.

[23] Department of Health and Social Care. Policy paper. The future of healthcare: our vision for digital, data and technology in health and care 2018.www.gov.uk/government/publications/the-future-of-healthcare-our-vision-for-digital-data-and-technology-in-health-and-care/the-future-of-healthcare-our-vision-for-digtial-data-and-technology-in-health-and-care. Accessed 19 May 2020.

[24] World Health Organisation. Draft Global Strategy on Digital Health 2020. http://www.who.int/docs/default-source/documents/gs4dhdaa2a9f352b0445bafbc79ca799dce4d.pdf?sfvrsn=f112ede5_38. Accessed 16 May 2020.

[25] Manrai A, Birgit H, Funke P (2016). Genetic misdiagnoses and the potential for health disparities. N Eng; J Med.

[26] Popejoy A, Fullerton S (2016). Genomics is failing on diversity. Nature..

[27] O'Neill O (2003). Some limits of informed consent. J Med Ethics.

[28] Pawson R, Greenhalgh T, Harvey G, et al. Realist synthesis: an introduction 2004. http://www.ccsr.ac.uk/methods/. Accessed 10 Feb 2020.

[29] Wong G. Data Gathering in realist reviews. Looking for needles in haystacks. In: Emmel, N, Greenhalgh, J, Manzano et al. Doing Realist Research London Sage. 2018. p.132.

[30] Pearson M. Realist synthesis: what is it and why might I want to use it? 2011. http://www.medicine.exeter.ac.uk/media/universityofexeter/medicalschool/research/pentag/20111-12-06_Pearson_Realist_review_What_How_Why.pdf. Accessed 15 Feb 2020.

[31] Pawson R, Greenhalgh T, Harvey G, Walshe K (2005). Realist review - a new method of systematic review designed for complex policy interventions. J Health Serv Res Policy.

[32] Pawson R (2006). Evidence-based policy: a realist perspective. Can J Prog Eval.

[33] Moher D (2015). Preferred Reporting Items for Systematic Review and Meta-Analysis Protocols (PRISMA-P) 2015 statement. Syst Rev.

[34] National Institute for Health Research. Research Champions 2020. http://www.nihr.ac.uk/patients-carers-and-the-public/i-want-to-help-with-research/research-champions.htm. Accessed 20 Apr 2020.

[35] Leigh-Hunt D. Identifying and managing internal and external stakeholder interests. Understanding organisations: identifying and managing internal and external stakeholder interests. Health Knowledge Education, CPD and Revalidation from Phast 2016. http://www.healthknowledge.org.uk/public-health-textbook/organisation-management/5b-understanding-ofs/managing-internal-external-stakeholders. Accessed 19 May 2020.

[36] Carrieri D, Pearson M, Mattick K, Papoutsi C, Briscoe S, Wong G, Jackson M (2020). Interventions to minimise doctors' mental ill-health and its impacts on the workforce and patient care: the Care Under Pressure realist review. Health Serv Deliv Res.

[37] INVOLVE Patient and public involvement in research and research ethics committee review 2009. http://www.invo.org.uk/wp-content/uploads/2011/12/INVOLVENRESfinalStatement310309.pdf. Accessed 12 Jun 2020.

[38] Wong G, Greenhalgh T, Westhorp G, Pawson R (2014). Development of methodological guidance, publication standards and training materials for realist and meta-narrative reviews: the RAMESES (Realist and Meta-narrative Evidence Syntheses - Evolving Standards) project. Health Serv Deliv Res.

[39] NCCfP. Online engagement: a guide to creating and running virtual meetings and events 2020. http://www.publicengagement.ac.uk/sites/default/files/publication/creating_and_running_virtual_events_-_april_2020_v1.pdf. Accessed 13 Apr 2020.

[40] Wong G, Westhorp G, Pawson R, Greenhalgh T Realist synthesis. Realist Training Materials. 2013. Available from: http://www.ramesesproject.org/media/Realist_reviews_training_materials.pdf. Accessed 01 Feb 2021.

[41] Hurwitz B, Greenhalgh T, Skultans V (2004). Narrative research in health and illness.

[42] Emmel N, Greenhalgh J, Manzano A (2018). Doing realist research.

[43] Rivas C, Vigours C, Cameron J, et al. A realist review of which advocacy interventions work for which abused women under what circumstances. Cochrane Database Syst Rev. 2019. 10.1002/14651858.CD013135. 10.1002/14651858.CD013135.pub2 PMC6598804 31254283

[44] Rycroft-Malone J, McCormack B, Hutchinson A. Realist synthesis: illustrating the method for implementation research. Impl Sci. 2012;7(1). 10.1186/1748-5908-7-33. 10.1186/1748-5908-7-33 PMC3514310 22515663

[45] Weetman K, Wong H, Scott E (2017). Improving best practice for patients receiving hospital discharge letters: a realist review protocol. BMJ Open.

[46] Mogre V, Scherpbier A, Dorman T. A realist review of educational interventions to improve the delivery of nutrition care by doctors and future doctors. Syst Rev. 2014. 10.1186/2046-4053-3-148. 10.1186/2046-4053-3-148 PMC4290450 25528058

[47] Ahikari B, Vincent R, Wong G. A realist review of community engagement with health research. Wellcome Open Res. 2019;4(87):1–35. 10.12688/wellcomeopenres.15298.2. 10.12688/wellcomeopenres.15298.1 PMC6611131 31289754

[48] Gough D. Weight of evidence: a framework for the appraisal of the quality and relevance of evidence. Res Pap Educ. 2007;22(2):213–28. 10.1080/02671520701296189.

