# Supplementary material for: The informed consent process in health research with under-served populations: a realist review protocol
Source: Syst Rev. 2021 Apr 9;10:103. doi: 10.1186/s13643-021-01652-2 (PMC8034278; doi:10.1186/s13643-021-01652-2)
Supplement: Supplementary file 1 — Additional file 1:. Draft MEDLINE search strategy [file 13643_2021_1652_MOESM1_ESM.doc]

**Additional File 1**

**Example Search strategy of database MEDLINE : identifying initial programme theories**

Database: Ovid MEDLINE(R) <1946 to May Week 3 2020>

Search Strategy:

--------------------------------------------------------------------------------

1 informed consent.mp. or exp Informed Consent/ (61481)

2 under served.mp. (308)

3 Health Services Research/ or health research.mp. or Biomedical Research/ (116591)

4 1 and 3 (2803)

5 under-served.mp. or exp Vulnerable Populations/ (10573)

6 4 and 5 (106)
